# Resting-state electroencephalography microstates as a marker of photosensitivity in juvenile myoclonic epilepsy

**DOI:** 10.1093/braincomms/fcae054

**Published:** 2024-02-19

**Authors:** Adolfo Mazzeo, Emanuele Cerulli Irelli, Giorgio Leodori, Marco Mancuso, Alessandra Morano, Anna Teresa Giallonardo, Carlo Di Bonaventura

**Affiliations:** Department of Human Neurosciences, Sapienza University, Rome 00185, Italy; Department of Human Neurosciences, Sapienza University, Rome 00185, Italy; Department of Human Neurosciences, Sapienza University, Rome 00185, Italy; IRCCS Neuromed, Pozzilli 86077, Italy; Department of Human Neurosciences, Sapienza University, Rome 00185, Italy; Department of Human Neurosciences, Sapienza University, Rome 00185, Italy; Department of Human Neurosciences, Sapienza University, Rome 00185, Italy; Department of Human Neurosciences, Sapienza University, Rome 00185, Italy

**Keywords:** resting-state EEG, idiopathic generalized epilepsy, photoparoxysmal response, visual network, Microstate B

## Abstract

Juvenile myoclonic epilepsy is an idiopathic generalized epilepsy syndrome associated with photosensitivity in approximately 30–40% of cases. Microstates consist of a brief period of time during which the topography of the whole resting-state electroencephalography (EEG) signal is characterized by a specific configuration. Previous neurophysiological and neuroimaging studies have suggested that Microstate B may represent activity within the visual network. In this case-control study, we aimed to investigate whether anatomical and functional alterations in the visual network observed in individuals with photosensitivity could lead to changes in Microstate B dynamics in photosensitive patients with juvenile myoclonic epilepsy. Resting-state electroencephalography microstate analysis was performed on 28 patients with juvenile myoclonic epilepsy. Of these, 15 patients exhibited photosensitivity, while the remaining 13 served as non-photosensitive controls. The two groups were carefully matched in terms of age, sex, seizure control and anti-seizure medications. Multivariate analysis of variance and repeated-measures analysis of variance were performed to assess significant differences in microstate metrics and syntax between the photosensitive and the non-photosensitive group. *Post hoc* false discovery rate adjusted unpaired *t*-tests were used to determine differences in specific microstate classes between the two groups. The four classical microstates (Classes A, B, C and D) accounted for 72.8% of the total electroencephalography signal variance in the photosensitive group and 75.64% in the non-photosensitive group. Multivariate analysis of variance revealed a statistically significant class–group interaction on microstate temporal metrics (*P* = 0.021). False discovery rate adjusted univariate analyses of variance indicated a significant class–group interaction for both mean occurrence (*P* = 0.002) and coverage (*P* = 0.03), but not for mean duration (*P* = 0.14). *Post hoc* false discovery rate adjusted unpaired *t*-tests showed significantly higher coverage (*P* = 0.02) and occurrence (*P* = 0.04) of Microstate B in photosensitive patients compared with non-photosensitive participants, along with an increased probability of transitioning from Microstates C (*P* = 0.04) and D (*P* = 0.02) to Microstate B. No significant differences were found concerning the other microstate classes between the two groups. Our study provides novel insights on resting-state electroencephalography microstate dynamics underlying photosensitivity in patients with juvenile myoclonic epilepsy. The increased representation of Microstate B in these patients might reflect the resting-state overactivation of the visual system underlying photosensitivity. Further research is warranted to investigate microstate dynamics in other photosensitive epilepsy syndromes.

## Teaser

Using resting-state electroencephalography microstates analysis, Mazzeo *et al*. revealed a significant difference in Microstate B metrics among juvenile myoclonic epilepsy patients with and without photosensitivity. These findings might reflect the resting-state overactivation of the visual system underlying photosensitivity and provide novel insights into the microstate dynamics in this condition.

## Graphical Abstract

**Graphical Abstract:**
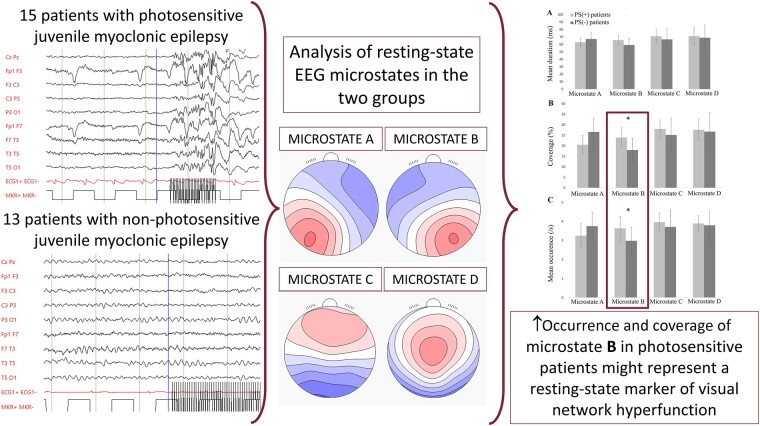

## Introduction

Juvenile myoclonic epilepsy (JME) is a type of idiopathic generalized epilepsy characterized by the occurrence of myoclonic seizures, often associated with generalized tonic–clonic seizures and, less commonly, absence seizures. It represents approximately 9% of all epilepsies and 18% of all idiopathic generalized epilepsies.^1,2^ JME typically begins during adolescence, although it can manifest between 8 and 40 years of age. Mandatory for its diagnosis is the presence of myoclonic seizures, usually within the first hour of awakening, associated with generalized polyspike–wave discharge and spike–wave discharge at a frequency of 3–5.5 Hz on the EEG.^3^

Photosensitivity (PS) is a common feature of idiopathic generalized epilepsies, especially in JME, where it is estimated to be present in approximately 30–40% of patients, although the reported range varies depending on different protocols used for intermittent photic stimulations.^4^ Neurophysiological investigations including EEG, magnetoencephalography and transcranial magnetic stimulation, along with MRI techniques have provided insights into the pathophysiology of PS. It is believed that PS results from a failure of the physiological inhibitory mechanisms happening during the excitation of the visual system due to structural and functional alterations within this network.^5,6^ Previous studies showed an increased thickness of the occipital and frontoparietal cortices in photosensitive [PS(+)] patients, along with a higher connectivity between the occipital cortex and supplementary motor area.^7-9^ EEG combined with functional MRI (fMRI) performed during photoparoxysmal response (PPR) in patients with JME has revealed early activation of the putamen and primary sensorimotor cortex, followed by a deactivation in the same structures as well as in the thalami and caudates.^10^ Additionally, increased resting-state connectivity has been observed between the pulvinar and the occipital, sensory-motor, anterior cingulate and supplementary motor cortices, which may explain the ‘visuomotor outflow’ originating myoclonus.^9,11^ These findings, altogether, point to recognize the origin of PS in an ictogenic network involving the striato-thalamocortical system.^12^ Furthermore, neuropsychological studies have demonstrated an overactivity of the visual system in individuals with PS, as PS(+) patients tend to exhibit a higher analytic score for the visual sensory modality.^13^

Microstates are a neurophysiological construct obtained from scalp EEG signal, reflecting the ‘atoms of thought’. They consist of brief periods of time (60–150 ms) in which the topography of the EEG signal is dominated by a specific configuration, remaining semi-stable. Quite consistently in the last 20 years, across different ages and conditions, four microstates have been identified (A–D). By means of fMRI, the source of microstates has been identified in the resting-state networks, each pertaining anatomo-functionally to distinct cortex areas and deep nucleus structures. Specifically, Microstate A has been attributed to the auditory/phonological network, Microstate B to the visual network, Microstate C to the salience network, and Microstate D to the attention network.^14,15^

The objective of our study was to investigate the characteristics of microstates in PS(+) JME patients and determine whether the previously identified alterations within the visual system could lead to changes in the resting-state microstates among this population. Given the correlation between Microstate B and the visual network, we hypothesized that PS(+) JME patients would exhibit an altered representation of Microstate B compared with non-photosensitive [PS(−)] JME patients. This would suggest a potential impact of PS on the underlying resting-state neural dynamics and functional organization of the visual network in individuals with JME.

## Materials and methods

### Participants

In this retrospective case-control study, the clinical charts and video EEG of patients followed in the Epilepsy Unit of Policlinico Umberto I from 1980 to 2022 were retrospectively reviewed by two experienced epileptologists (A.T.G. and E.C.I.). Patients with JME were selected according to the diagnostic criteria for JME: myoclonic jerks mostly occurring on awakening, facilitated by sleep deprivation and stress; onset age between 6 and 25 years; no intellectual disability; and an EEG showing a normal background with at least one polyspike–wave discharge/spike–wave discharge.^16^

Inclusion criteria were the following: (i) diagnosis of JME according to the above-mentioned criteria, (ii) presence of PPR induced by intermittent photic stimulation and (iii) availability of the EEG data. The photosensitive group comprised 15 patients. They were matched with 13 PS(−) JME patients based on age, sex, seizure control and anti-seizure medication (ASM) used at the time of the EEG. The PS(−) group comprised individuals who had never displayed PPR in their previous EEGs and had no history of photosensitive seizures, pattern sensitivity or eye closure sensitivity. Demographic and electroclinical characteristics of the groups are reported in Table 1.

**Table 1:** Demographic and clinical characteristics of JME patients according to PS status

	PS(+) patients (*n* = 15)	PS(−) patients (*n* = 13)	*P*-value
Age at EEG time, years, median (IQR)	22 (19–29)	22 (20–29)	0.9
Female sex, *n* (%)	11 (73.3)	9 (69.2)	0.8
Family history of epilepsy in a 1st or 2nd degree relative, *n* (%)	6 (40)	3 (23)	
Disease duration, years, median (IQR)	11 (7–17)	6 (5–15)	
Seizure freedom duration at EEG time, months, median (IQR)	60 (30–78)	36 (24–72)	0.38
Myoclonus occurrence during PPR, *n* (%)	8 (53.3)		
Waltz grade			
Grade I/II, *n* (%)	3 (20)		
Grade III/IV, *n* (%)	12 (80)		
ASM used in monotherapy at EEG time			
Levetiracetam, *n* (%)	6 (40)	5 (38.5)	0.9
Valproic acid, *n* (%)	7 (46.7)	6 (46.1)	0.9
Phenobarbital, *n* (%)	2 (13.3)	2 (15.4)	0.9

ASM, anti-seizure medication; EEG, electroencephalography; IQR, interquartile range; JME, juvenile myoclonic epilepsy; PPR, photoparoxysmal response; PS, photosensitivity.

The study was approved by the local ethics committee and informed consent was obtained by all participants.

### EEG acquisition and pre-processing

Video EEG data were recorded using the Xltech System (Oakville, Canada), 21 channels, International 10–20 system. The reference was placed on FPz and the ground on FCz. Impedance was kept below 5 kΩ for all electrodes. Electrophysiological data were continuously recorded with a bandwidth of 0.05–100 Hz and sampled at a rate of 1000 Hz. During the EEG recording, the subjects stayed awake, with eyes closed, seated in a comfortable chair in a silent room, for 20 min. Intermittent photic stimulation was performed according to the European consensus.^17^ PPR was classified according to Waltz patterns^18^ and video EEGs were inspected to identify the presence of myoclonus during PPR.

### Microstate analysis

The raw EEG data were processed using the EEGLAB toolbox (v.2022.1)^19^ running on MATLAB. Data were first resampled at 250 Hz and bandpass filtered 2–20 Hz using a finite impulse response filter with 2000 filter coefficients as suggested for microstates analysis.^20,21^ The data were subsequently re-referenced to the average reference. Epileptiform discharge-free epochs were selected by an experienced epileptologist (E.C.I.), and artefact removal was eventually performed using the clean_artifacts function from the Cleanline plugin,^22^ which identifies and removes various types of artefacts, such as flatline channels, noisy channels and temporal bursts. The algorithm employs a combination of criteria, including flatline, channel, line noise, burst and window criteria, to detect and correct for artefacts. Channels with a high proportion of bad data (more than 60% of recording time) were removed from the analysis and interpolated using spherical interpolation. Residual blinks, muscle activity and electrical noise artefacts were rejected by independent component analysis (ICA) using the FastICA algorithm. After component rejection, the cleaned data were saved for microstate analysis.

Microstate analysis was conducted using the Microstate toolbox.^23^ The toolbox performs clustering and segmentation of the EEG data into microstates. Clustering parameters were set to include a minimum of four and a maximum of six microstate classes, with a focus on global field power peaks and polarity independence. A modified k-means algorithm was employed for clustering, and the best solution was selected based on the highest explained variance. The individual and grand mean microstate classes were automatically sorted according to previously published templates.^24^

We extracted a variety of temporal parameters for each of the classic A, B, C and D microstates according to previously described methods.^25^ First, we calculated the explained variance, which represents the proportion of total data variance explained by a specific microstate. Next, we computed the mean duration, computed as the average time in milliseconds that a microstate is active, and the mean occurrence, which refers to the frequency at which a specific microstate appears. Lastly, we determined the coverage, a measure indicating the percentage of time each microstate class accounts for within the entire analysis period, expressed as a ratio of 1.

Following this, we further investigated the temporal dynamics of microstates by examining the syntax or the transition probabilities between microstates. To assess the syntax, we computed the transition percentages, which are the normalized counts of transitions from one microstate class to another. We then calculated the expected transition probabilities under the assumption of random transitions, determined by the relative occurrences of each microstate. The difference between the observed and expected transitions, defined as Delta transition, was also evaluated to discern the degree to which transitions between specific microstates were driven by patterns beyond chance. These analyses identified non-random sequential relationships and dynamics between different brain microstates.

### Statistical analysis

We assessed the effects of group and class separately on microstate topographic distribution and temporal features by using a topographic ANOVA (TANOVA) and a multivariate ANOVA (MANOVA), respectively.

To investigate systematic differences in the spatial distribution of microstate class maps across groups we employed the Ragu toolbox performing TANOVA, a non-parametric statistical test designed specifically for analysing differences in scalp potential field distributions using global randomization statistics.^26^ Briefly, it employs a global measure of scalp field differences (***s***) and assesses the similarity of the topography between groups and classes. With TANOVA, we tested the significance of class and group effects by comparing the value of ***s*** obtained with the real data against a distribution generated from a permutation-based resampling procedure (1000 permutations). Each individual scalp field of each condition was normalized before the TANOVA by scaling to unity variance.

A two-way MANOVA was run to determine the effect of group [PS(+) and PS(−)] and class (A, B, C and D) on microstates’ temporal metrics: mean duration, mean occurrence and coverage (combined dependent variables). Therefore, false discovery rate (FDR)-adjusted repeated measures ANOVAs (one for each microstate temporal parameter) were performed using group as a between-participants factor [PS(+) patients versus PS(−) patients] and microstate class (A versus B versus C versus D) as a within-participants factor. *Post hoc* comparison was performed through a FDR-adjusted unpaired *t*-test.

For microstate classes showing significant between-group differences, we also compared Delta transition between groups through a FDR-adjusted unpaired *t*-test. Additionally, we performed a correlation analysis to evaluate possible associations between microstate parameters and clinical variables, including Waltz grade or the occurrence of body myoclonia during PPR.

Finally, receiver operative curve analysis was used to evaluate the ability of significant microstate parameters to discriminate between PS(+) and PS(−) patients.

MANOVA, ANOVA and unpaired *t*-test were performed using SPSS version 25. All statistical analyses were performed using a significance level set at *P* < 0.05.

## Results

The identified microstates accounted for 72.8% of the total EEG signal variance in the PS(+) group and 75.64% in the PS(−) group, with no significant differences observed between the groups (*P* = 0.17).

**Figure 1:**
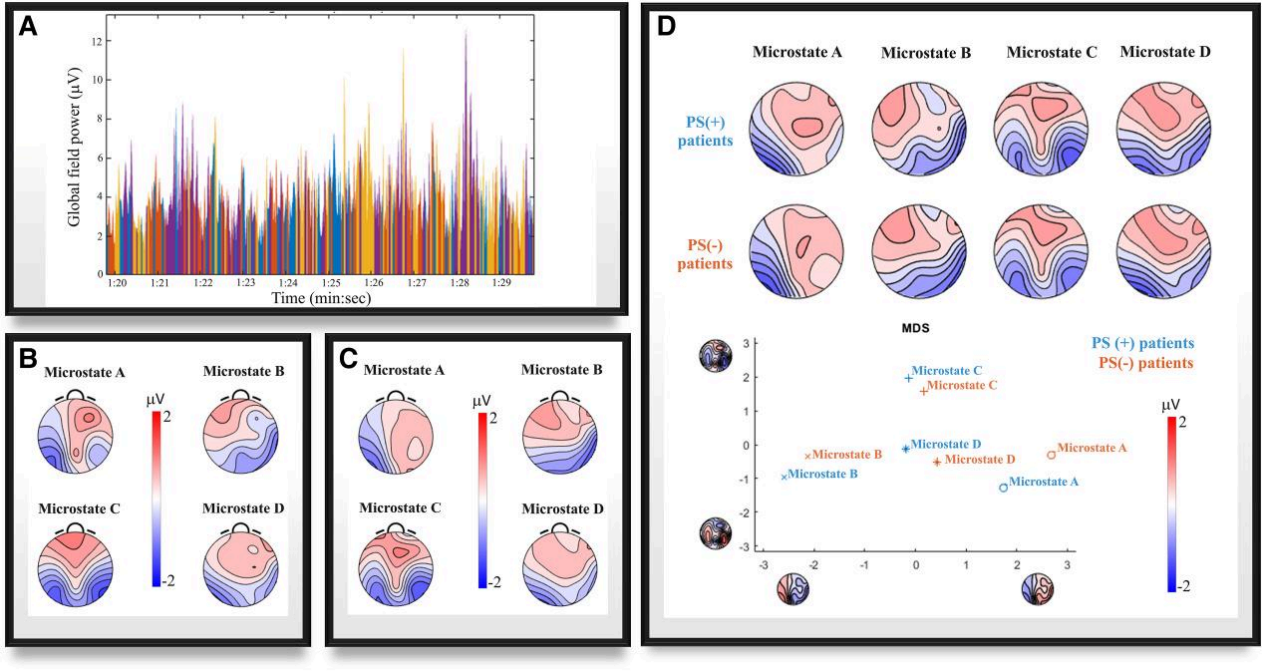
**Global field power and microstate topographic maps.** Panel **A** shows global field power time series over a 10-s interval from one exemplificative patient. Each presented peak indicates a distinct topographic map. Panel **B** indicates microstate maps from one exemplificative participant. Panel **C** shows grand-grand-average microstate class maps across subjects and groups. In the upper part of Panel **D**, microstate topographic maps are shown according to PS status. In the lower part of Panel **D**, a multidimensional scaling analysis is displayed, to allow a spatial comparison for each microstate between the two groups. Multidimensional scaling analysis project points from a multidimensional space into a lower-dimensional space, by subjecting the spatial principal component analysis to the average group maps, enabling the visualization of the data. The maps shown on the *x*- and *y*-axes represent principal component analysis eigenvector maps. Graph points representing groups with similar topographies are depicted in close proximity to each other. MDS, multidimensional scaling; PS(+), photosensitive patients; PS(−), non-photosensitive patients.

The temporal parameters of microstates according to PS status are reported in Table 2. Figure 1A shows global field power time series whereas Fig. 1B indicates microstate maps from one exemplificative participant. Figure 1C shows grand-grand-average microstate class maps across subjects and groups. In Fig. 1D, microstate topographic maps are shown according to PS status.

**Table 2:** Microstate temporal parameters in juvenile myoclonic patients according to photosensitivity status

	PS(+) patients (*n* = 15), mean ± SD	PS(−) patients (*n* = 13), mean ± SD	*P* value	FDR-adjusted *P* value
Mean duration (ms)				
Microstate A	62.91 ± 6.07	68.18 ± 8.22	0.128	0.26
Microstate B	65.73 ± 6.17	59.17 ± 9.53	0.038	0.15
Microstate C	79.96 ± 9.55	66.72 ± 14.88	0.371	0.49
Microstate D	71.11 ± 12.20	69.00 ± 17.45	0.711	0.71
Mean occurrence (Hz)				
Microstate A	3.25 ± 0.64	3.75 ± 0.75	0.066	0.13
Microstate B	3.72 ± 0.62	2.98 ± 0.72	0.007	0.04*
Microstate C	3.96 ± 0.48	3.71 ± 0.88	0.352	0.47
Microstate D	3.89 ± 0.41	3.80 ± 0.99	0.731	0.73
Coverage (%)				
Microstate A	20.48 ± 4.38	30.14 ± 17.07	0.044	0.09
Microstate B	24.48 ± 4.44	17.98 ± 5.65	0.002	0.02*
Microstate C	28.00 ± 4.31	25.15 ± 8.01	0.243	0.32
Microstate D	27.61 ± 5.02	26.73 ± 9.26	0.754	0.75

FDR, false discovery rate; JME, juvenile myoclonic epilepsy; ms, milliseconds; PS(+), photosensitive patients; PS(−), non-photosensitive patients; SD, standard deviation.

^*^Indicates statistically significant variables after FDR adjustment.

TANOVA results revealed a significant main effect of class (*P* = 0.001) and non-significant main effect of group (*P* = 0.167) and group–class interaction (*P* = 0.605) on microstate topographic distribution (Fig. 1D).

MANOVA showed a statistically significant class–group interaction on the combined dependent variables [*F*(9, 185) = 2.24, *P* = 0.021; Wilks’ *Λ* = 0.778]. Therefore, FDR-adjusted univariate ANOVAs revealed a significant class–group interaction for both occurrence [*F*(3, 78) = 5.51, *P* = 0.002] and coverage [*F*(3, 78) = 3.61, *P* = 0.03], but not for duration [*F*(3, 78) = 2.22, *P* = 0.14]. The same ANOVAs revealed a significant effect for class on occurrence [*F*(3, 78) = 6.59, *P* = 0.001], but not on duration [*F*(3, 78) = 1.18, *P* = 0.29] and coverage [*F*(3, 78) = 2.5, *P* = 0.12], whereas the group had no significant effect on any microstate characteristics.

**Figure 2:**
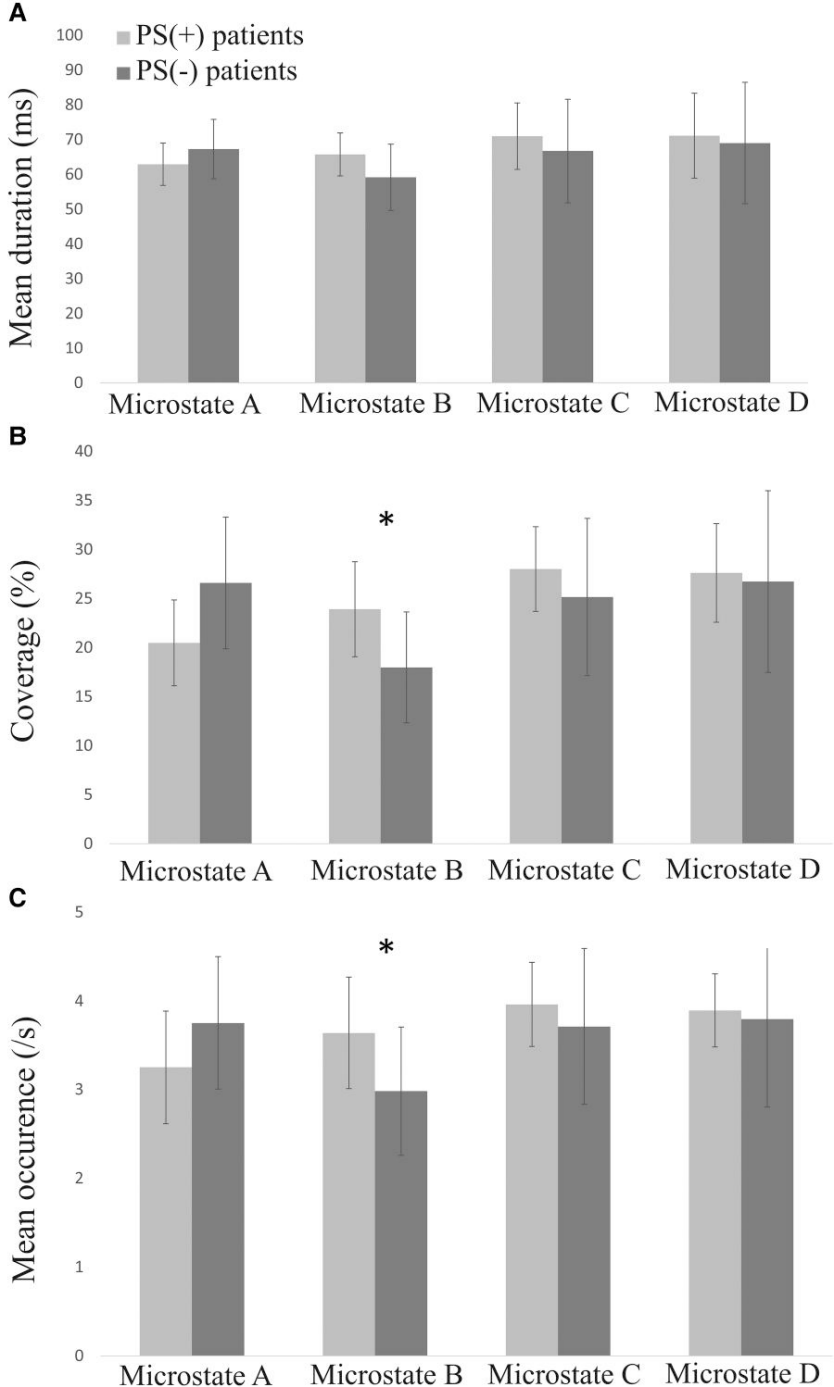
**Temporal metrics of microstate classes according to photosensitivity status.** Repeated-measures ANOVAs were performed for mean duration (**A**), coverage (**B**) and mean occurrence (**C**). A significant class–group interaction was found through repeated-measures ANOVA for microstate occurrence [*F*(3, 78) = 6 5.50, *P* = 0.002] and coverage [*F*(3, 78) = 3.61, *P* = 0.03]. *Post hoc* unpaired *t*-tests adjusted for FDR revealed a significant difference (indicated by asterisks in the figure) for both Microstate B occurrence [*t*(26) = 2,57, *P* = 0.04)] and coverage [*t*(26) = 3.01, *P* = 0.02)]. PS(+), photosensitive patients; PS(−), non-photosensitive patients.

*Post hoc* FDR-adjusted unpaired *t*-tests demonstrated that Microstate B covered significantly more time in PS(+) patients than in PS(−) individuals (*P* = 0.02) while no significant differences were observed for the other microstate coverage. Furthermore, FDR-adjusted unpaired *t*-tests indicated that Microstate B was significantly more frequent in PS(+) patients than in PS(−) patients (*P* = 0.04). No significant differences were found for the other classes (Fig. 2).

The receiver operative curve analysis indicated that Microstate B occurrence could discriminate PS(+) patients from PS(−) patients with good accuracy (area under the curve = 0.78, 95% confidence interval 0.58–0.94, *P* = 0.01; sensitivity = 73.3%, specificity = 84.6%, cut-off = 3.38 Hz), as well as Microstate B coverage (area under the curve = 0.85, 95% confidence interval 0.66–0.99, *P* = 0.002; sensitivity = 86.7%, specificity = 84.6%, cut-off = 21.63%).

Considering the statistical significance difference found for Microstate B, the transition probability from Microstate B to the other microstates was analysed. FDR-adjusted unpaired *t*-tests revealed that PS(+) patients showed significantly more transitions from Microstates C to B (*P* = 0.04) and from Microstates D to B (*P* = 0.02) when compared with PS(−) patients.

Assessing possible correlations between microstate parameters and clinical characteristics, after adjusting for FDR, no significant differences were observed between the temporal parameters of the four microstates and Waltz grade or the occurrence of body myoclonia during PPR.

## Discussion

To our knowledge, this is the first study investigating resting-state EEG microstates in PS(+) patients to assess whether a peculiar microstate signature might represent a potential marker of PS. As hypothesized, our study reveals a different microstate profile at rest in a population of JME PS(+) patients compared with PS(−) patients, after controlling for ASM burden, seizure control and other demographic characteristics. Notably, we purposefully matched patients for JME syndrome, in order to selectively focus on the impact of the PS trait on microstate dynamics and avoid the effect of other syndrome-related factors.

Specifically, in PS(+) patients, we identified (i) an increased occurrence and coverage of Microstate B compared with PS(−) patients and (ii) an increased probability of transitioning from Microstates C and D to Microstate B.

In the last years, the role of microstates in neuroscience has grown remarkably, and there is evidence that they might be used as biomarkers of several conditions, such as schizophrenia, 22q11.2 deletion, Alzheimer’s disease, frontotemporal dementia and migraine.^27-30^ Moreover, preliminary data have also highlighted the possible relevance of microstate metrics in some epilepsy syndromes.^31,32^

In this study, we observed an increased occurrence and coverage of Microstate B in PS(+) patients, along with a higher probability of transitioning into Microstate B from other microstate classes. Previous fMRI studies have consistently linked Microstate B with the activity of the extrastriate visual areas bilaterally.^15,33,34^ Moreover, a high-density EEG study demonstrated that Microstate B occurrence and coverage increase during eyes-open wakeful rest compared with the eyes-closed condition.^35^

Occurrence and time coverage of a specific microstate are thought to mirror the tendency of its neural substrates to activate and, therefore, the relative time during which that network is predominant at rest.^25^ Therefore, an over-representation of Microstate B in PS(+) patients might signify that in this population, there is higher tendency of the visual network to be activated at rest as well as an increased susceptibility of this system to hyper-activate when exposed to visual stimuli. This microstate trait could therefore represent a potential signature of the anatomical and functional alterations of the visual network thought to be pathophysiological features of PS.

Indeed, our results are in line with previous findings from transcranial magnetic stimulation, MRI and fMRI studies, which have highlighted the pivotal role of the hyper-excitability and hyper-connectivity of the visual system in the generation of PS.^5,7-11^ As microstates are thought to be driven mainly by alpha oscillations,^36^ our finding of a different profile of microstates in PS(+) patients might reflect the decreased inhibition of alpha rhythm generating networks shown in PS epilepsy by means of fMRI.^9^

Furthermore, the overactivation of the rest of the visual system might also explain the peculiar neuropsychological traits observed in PS(+) patients, who have been shown to have different visual information processing skills.^13^ Indeed, previous studies conducted on healthy subjects indicated that Microstate B might be related to the visuospatial attention and processing aspects of fluid intelligence.^37-39^

Accordingly, an increased representation of Microstate B has been also identified in patients affected by migraine without aura. In these patients, hyperactivity of the visual system is indeed considered a trait of the disease,^30^ and there is plenty of evidence pointing to consider migraine, epilepsy and PS as being part of the same continuum.^40^

Interestingly, a previous study conducted in patients with temporal lobe epilepsy has shown that levetiracetam induces a reduction in Microstate B duration and occurrence.^41^ Since levetiracetam is extremely efficacious in suppressing PPR^42,43^ and considering our findings, it could be argued that the changes of Microstate B induced by levetiracetam might reflect the inhibition of the hyper-excitability of the visual system. If confirmed, Microstate B temporal metrics could therefore potentially serve as biomarkers to assess the efficacy of ASMs on PS. Clearly, further studies are necessary to evaluate microstate modulation by other ASMs and the role of Microstate B as a marker of PS.

Finally, after correcting for FDR, an exploratory analysis revealed no significant differences in microstate metrics among patients with different Waltz grades or in those patients with body myoclonia occurring during PPR. However, the small number of patients with motor phenomena (8/15), as well as the limited number of patients with I/II Waltz pattern (3/15), might have undermined the power of our analysis to detect differences in the PS(+) group. Further studies are necessary to provide a more comprehensive understanding of microstates among PS(+) patients with different clinical characteristics.

Our study has some limitations, including the retrospective design, which may have implied some sampling bias, and the relatively small sample size, which may have underpowered our analysis on between-group differences. The use of 19-channel EEG may have limited the spatial resolution of our microstate analysis, even if previous studies have shown microstates to be quite consistent even when extrapolated from low-density EEG.^14^ Furthermore, although we matched patients for ASM, we could not definitively exclude their effect on microstate metrics. Finally, although the selective inclusion of JME patients in this study might prevent us from generalizing our findings in PS(+) patients with other epilepsy syndromes, our methodological approach helped us to specifically capture the effect of PS on microstate dynamics. Indeed, several studies demonstrated significant differences in terms of resting-state networks between different idiopathic generalized epilepsy syndromes potentially associated with PS, and their inclusion might have strongly affected our analysis on microstates.^44^

In conclusion, our study provides novel insights on resting-state EEG microstate dynamics underlying PS in patients with JME. The increased representation of Microstate B in these patients might reflect the resting-state overactivation of the visual system underlying PS. Further studies are needed to confirm our data and investigate microstate dynamics in PS(+) patients with other epilepsy syndromes.

## Data Availability

De-identified data are available upon reasonable request.
