# Correction to: Resting-state electroencephalography microstates as a marker of photosensitivity in juvenile myoclonic epilepsy

**DOI:** 10.1093/braincomms/fcae125

**Published:** 2024-04-18

This is a correction to: Adolfo Mazzeo, Emanuele Cerulli Irelli, Giorgio Leodori, Marco Mancuso, Alessandra Morano, Anna Teresa Giallonardo, Carlo Di Bonaventura, Resting-state electroencephalography microstates as a marker of photosensitivity in juvenile myoclonic epilepsy, *Brain Communications*, Volume 6, Issue 2, 2024, fcae054, https://doi.org/10.1093/braincomms/fcae054

During production of the original version of this paper, EEG was changed to “electroencephalography” in the abstract and keywords. This has now been corrected back to “EEG”.

